# The Growing Use of Copy-Paste and Its Impact on Creation and Propagation of Documentation Errors

**DOI:** 10.1016/j.chpulm.2026.100259

**Published:** 2026-04-12

**Authors:** Ryan Grattan, Sarah T. Florig, Tanuj Devra, Vishnu Mohan, Jeffrey A. Gold

**Affiliations:** a Division of Informatics, Clinical Epidemiology and Translational Data Science, OHSU, Portland, OR

**Keywords:** administration retrospective studies, copy-paste, critical care, cross-sectional studies, documentation, electronic health records, humans inpatients organization

## Abstract

**Background:**

Electronic health records have introduced tools to improve administrative productivity, but they also pose challenges that may affect documentation accuracy and clinical decisions, especially in high-acuity patients.

**Research Question:**

What is the prevalence and evolution of copy-paste practices in daily progress notes within an ICU, and how do these practices influence the creation or propagation of documentation errors?

**Study Design and Methods:**

This retrospective cohort study analyzed progress notes written by residents and attendings in a medical ICU at a single academic center from 2017 to 2022, focusing on the use of copied content. A random sample of 180 resident notes was manually reviewed for clinical accuracy. Content sources and copy-related errors were examined for correlations with encounter characteristics and documentation burden.

**Results:**

At total of 60,127 notes were analyzed for composition trends. Over the study period, resident notes lengthened by 21%, with copied content increasing by 34% and direct and templated content decreasing by 52% and 9%, respectively. Attending note length increased by 2%, with no change in copied content. For residents, periods with more notes written per day correlated positively with note length, and proportion of templated and copied text but negatively correlated with direct text. Chart review of resident notes revealed copied text in all samples; one-half were from another author. A total of 521 documentation errors were identified, with errors of omission being the most common (61%), then commission (28%), and temporal errors (11%). Temporal errors correlated with elapsed stay and copied text.

**Interpretation:**

In our medical ICU, progress notes are getting longer, and longer notes are associated with increased use of passive documentation such as copied content. For residents, this behavior is exacerbated during times of increased documentation burden. Copied content introduces a mechanism for errors to be created and propagated, as evidenced by the documentation errors related to copy-paste identified in this study.

## Graphical abstract

**Figure:**
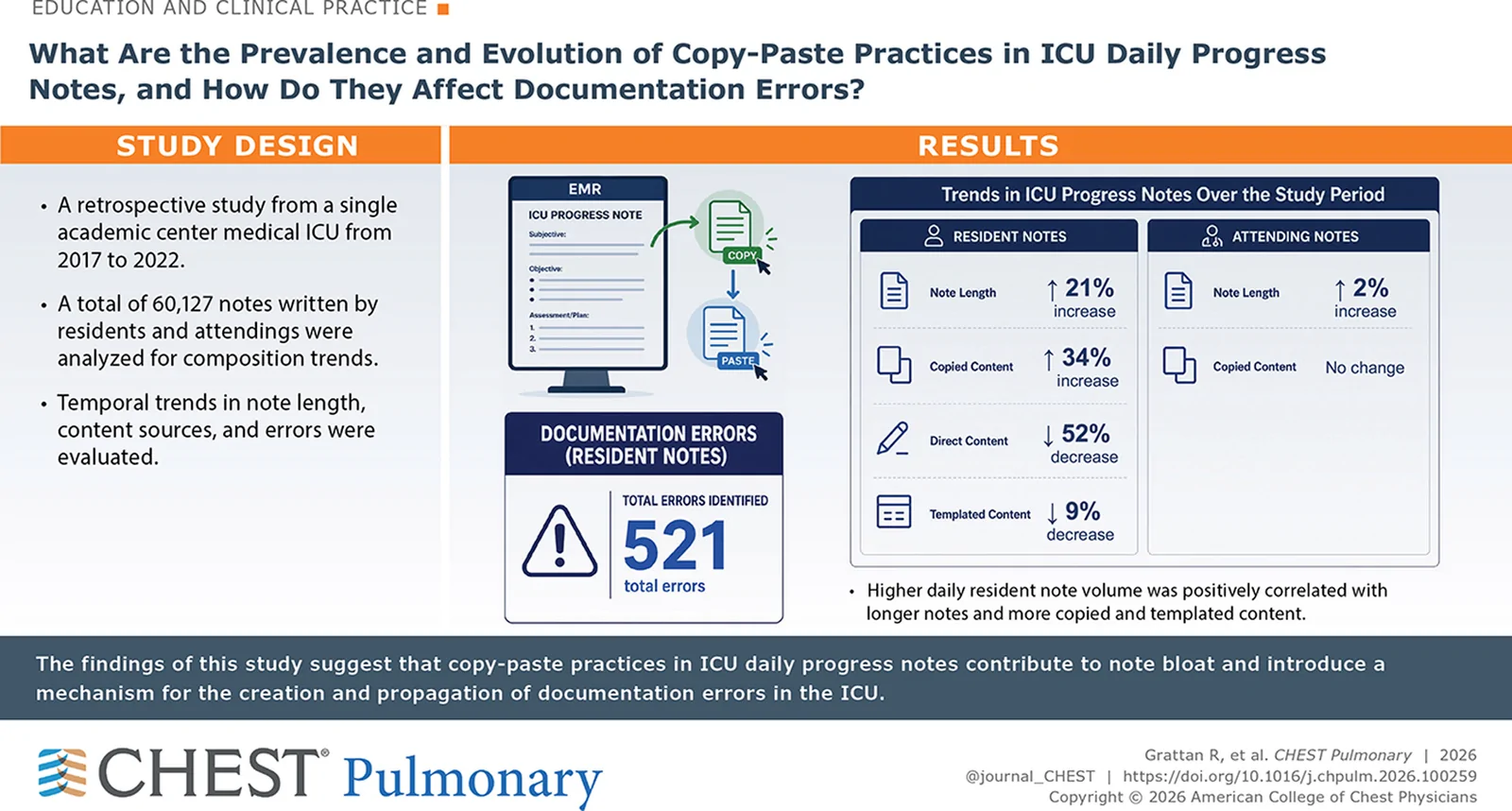

**Take-Home Points**

**Study Question:** What is the prevalence and evolution of copy-paste practices in daily progress notes within an ICU, and how do these practices influence the creation or propagation of documentation errors?

**Results:** We found that progress notes grew in length predominantly due to an increasing use of copied content, this behavior is exacerbated by periods of higher documentation burden, and 86% of notes contained at least 1 error related to copy-pasted content.

**Interpretation:** This study demonstrates that copy-paste is a contributor to note bloat and introduces a mechanism for the creation and propagation of errors in the ICU.

The adoption of electronic health records (EHRs) has enhanced clinical documentation by improving access to patient records, streamlining document organization, and promoting more complete data entry.^1^^,^^2^ However, EHRs have increased provider time for clinical documentation, often at the expense of direct patient care.^3^ To reduce this burden, clinicians increasingly rely on passive content creation tools such as copy-paste functions or templated text. These practices have contributed to note bloat, lengthy notes that can obscure key pieces of information.^4^ Prior studies demonstrate both an increase in note length and the proportion of duplicated text, with estimates that 50% to 78% of note text is duplicated and that this proportion is growing over time.^5^, ^6^, ^7^, ^8^, ^9^, ^10^

Increased text duplication and note length have been associated with multiple downstream harms. Threats to the quality and accuracy of medical documentation are significant patient safety concerns. Previous research has raised concerns about copy-paste and the loss of clinical narrative, obfuscation of timely clinical details due to information scatter or increased document length, and increased risk of documentation errors.^10^, ^11^, ^12^ These errors can have real-world consequences; studies have demonstrated an association between high text duplication and hospital readmissions and hospital-acquired infections.^13^, ^14^, ^15^ Moreover, up to 25% of patients identify errors in their patient record—errors that are recognized contributors to EHR-related diagnostic error and broader safety concerns.^13^^,^^16^, ^17^, ^18^, ^19^, ^20^, ^21^

Certain populations, such as the critically ill receiving care in an ICU, may be particularly vulnerable to the drawbacks of copy-paste. The high clinical acuity of the ICU may exacerbate issues of information overload, scatter, and conflict.^12^ Greater data volumes per patient, increased need for timely bedside care, and complex clinical courses make it challenging to capture an ICU encounter accurately and cohesively in clinical documentation. The goal of this study was to characterize the prevalence and evolution of copy-paste usage and its impact on error types in ICU documentation. It quantifies trends in content sources (eg, copied, templated, direct text), examines documentation behavior under changing documentation burden, and characterizes errors within copied text.

## Study Design and Methods

### Setting and Design

This retrospective cross-sectional analysis was performed in the medical ICU (MICU) of an academic medical center. The MICU is a closed ICU with > 90% of patients cared for by a dedicated primary service. The medical center was using Epic (Epic Systems Corp) for its EHR throughout the study period. This study follows the Strengthening the Reporting of Observational Studies in Epidemiology guideline for observational studies. This study was approved by the OHSU institutional review board (No. 17599), which granted a waiver of informed consent.

### Data

Descriptive data on daily progress notes written by MICU resident and attending physicians between January 1, 2017, and December 31, 2022, were extracted from Epic’s audit log data. Encounters with non-MICU authors were excluded from the study.

Epic classifies each character in a note into 12 categories that identify the originating source. Following prior work, these 12 categories were mapped to 3 broader content source categories,^5^^,^^22^ including copied, templated, and direct. Copied text refers to text that the user actively identified in other parts of the chart, selected and duplicated, and then included in the document being written (ie, copy-paste). Templated text is content that was derived by automated processes within EHRs, such as templates or Epic’s proprietary SmartLinks. Direct text is text that was either entered via typing on a keyboard or voice-dictated into the document by the note author.

A total of 180 notes written in 2022 were manually chart reviewed. This was achieved by randomly selecting 15 notes per month or roughly 3% of resident-written notes for 2022. This was considered to be the maximum feasible scope for a single-reviewer manual chart review. Given no statistically significant change in copied content and that the bulk of documentation is performed by residents in our MICU, a manual chart review of attending-written progress notes was not performed. Using features within Epic, copied text was highlighted while templated and direct text were masked. The date and time of final revision for the note were documented. Copied text was then manually assessed for errors by comparing with reference standards or primary data wherever possible, such as radiology reports, laboratory values, the Medication Administration Record, consultant-documented recommendations, or signed orders. If documentation differed from these reference standards within the 24-hour period before the final revision time of the note, an error was noted. These errors were categorized into errors of omission, errors of commission, or temporal errors.^19^^,^^23^, ^24^, ^25^ Errors of omission occurred when there was evidence of an action (eg, antibiotics were administered) but no documentation provided. Errors of commission occurred when there was evidence of an action (eg, heparin stopped on the Medication Administration Record), but documentation suggested an alternative action was performed (eg, continue heparin). Temporal errors occurred when a statement within copied text depicted a clinical scenario that was no longer accurate based on review of the reference standards. See Table 1 for reference standards, examples of documentation prompting review, and examples of omission, commission, and temporal categories that were identified during chart review.

**Table 1:** Examples of documentation and associated reference standard and resultant error type identified during chart review

Reference Standard	Example	Error Type
MAR	MAR shows an order in place for ampicillin 2g every 4 hours, but not documented in Plan	Omission
MAR	“holding quetiapine” documented, however MAR shows medication still ordered and being received	Commission
Lab Results, MAR	“ScVO2 improved today and stopping dobutamine” documented. However, ScVO2 declined since originally written and dobutamine restarted.	Temporal
Radiology Report	“No new imaging” documented (copy forwarded from note two days prior). Two radiographic studies done in last 24 hours to confirm central line and Dobhoff tube placement.	Commission
Consultant Recommendation	Wound care team following and leaving recommendations, however recommendations not integrated in Assessment/Plan	Omission
Signed Order	Vt 4-6 mL/kg; plateau pressures < 30” Two orders within 24 hour period before note that are inconsistent with Vt goal.	Commission

MAR = Medication Administration Record.

### Analysis

Temporal trends in note length and content sources (eg, templated, copied) were characterized. Note length was assessed by both median and total number of characters per note. Content sources were calculated as a proportion of total characters committed per note.

To assess predictors of note length and content source, we calculated the daily documentation burden for each resident, a known metric for this purpose.^26^ The number of notes written in a given day, and the minimum and maximum number of daily notes written by each author for any day they were on clinical service, were extracted. This was then used to generate an individualized, normalized score for the number of notes written on a given day by that author. This individualized score was developed to minimize the impact of differing documentation behaviors between authors.

To assess the impact of documentation burden on note-writing behavior, Spearman correlation coefficients were calculated between the measure of estimated documentation burden, the proportion of each content source category, and the total number of characters in a note.

Finally, data from the chart review were used to characterize error types, their location within the note, and authorship of copied content. Error types were correlated with length of stay, proportion of each content source, and total note length.

## Results

For the longitudinal trend analysis, 60,127 notes were identified from 6,313 unique patient encounters. The median note length was 3,723 characters (interquartile range [IQR], 1,502-9,575). Resident-written notes had a median length of 10,690 characters (IQR, 8,292-13,346), significantly longer than attending-written notes (*P* < .001), which had a median length of 1,922 characters (IQR, 1,236-4,549). For resident-written notes, 50% of text was copied, 34% was templated, and 16% was directly entered. For attending-written notes, 17% was copied, 57% was templated, and 26% was direct. See Table 2 for a summary of these results.

**Table 2:** Note length and proportion of sources of content for resident and attending notes

	Attending	Resident
**Median Note Length (# characters)**	1,922 (IQR 1,236 – 4,549)	10,690 (IQR 8,292 – 13,346)
**Proportion Copied Text**	17%	50%
**Proportion Templated Text**	57%	34%
**Proportion Direct Text**	26%	16%

**Figure 1:**
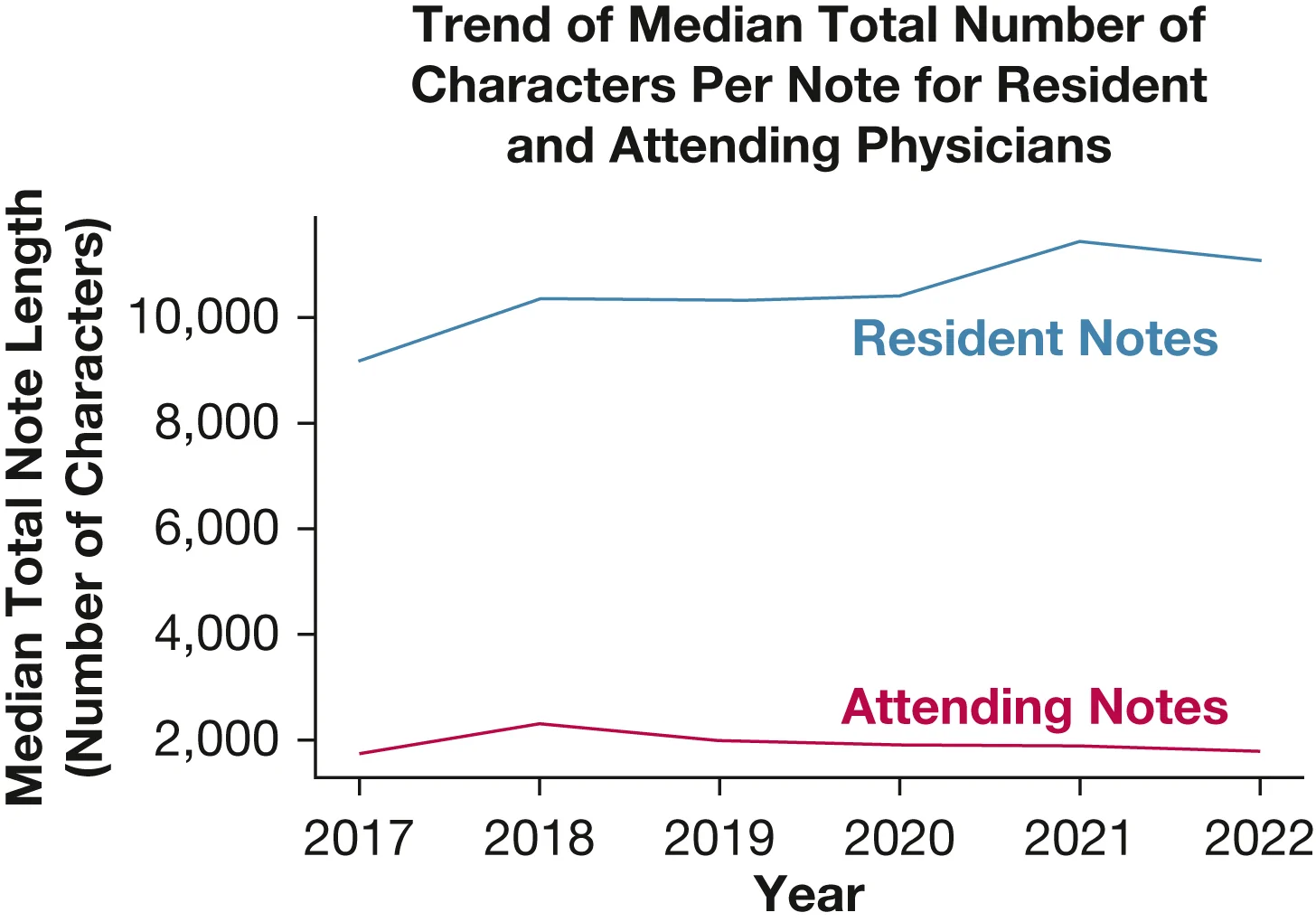
Annual median length of notes by resident (blue) and attending (red) physicians showing rising note length for resident physicians.

Between 2017 and 2022, resident progress note length increased from 9,196 characters (IQR, 6,957-11,298) to 11,092 characters (IQR, 8,699-13,828), a 21% increase (*P* < .001) (Fig 1). Attending progress note length rose modestly, from 1,775 (IQR, 1,210-5,568) to 1,807 (IQR, 1,806-2,801), a 2% increase (*P* < .001) (Fig 1).

**Figure 2:**
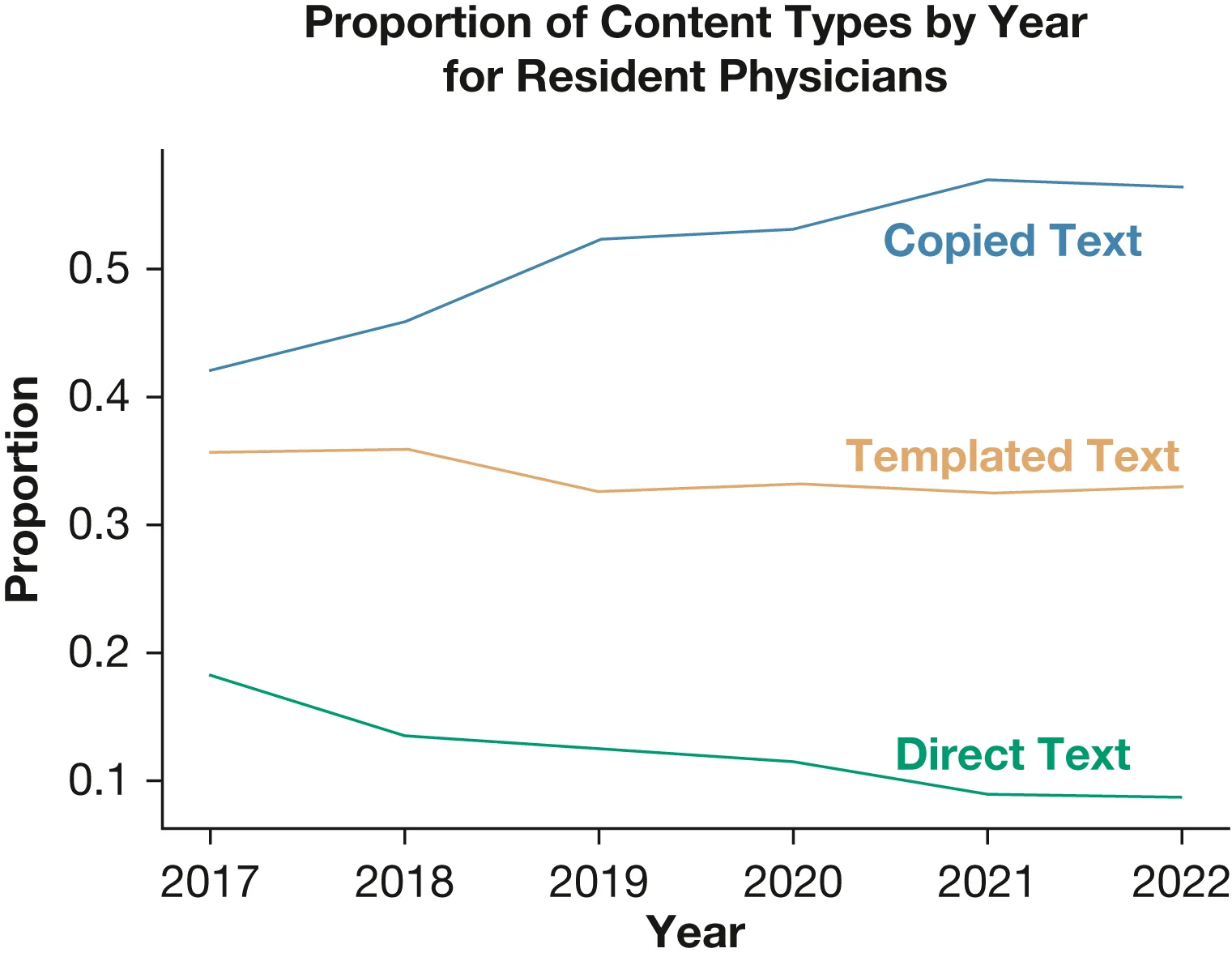
Proportion of copied, direct, or templated text in notes written by resident physicians. The proportion of copied text has increased, whereas both templated and direct text has decreased.

Residents’ notes showed an increase in copied text from 42% in 2017 to 56% in 2022 (33% relative increase; *P* < .001) (Fig 2), and declines in direct text (18% to 9%; relative decrease of 50%; *P* < .001) and templated text (36% to 33%; relative decrease of 8%; *P* < .001). Total note length was positively correlated with copied text proportion (ρ = 0.40; *P* < .001) and negatively correlated with templated text (ρ = −0.11; *P* < .001) and direct text (ρ = −0.36; *P* < .001).

**Figure 3:**
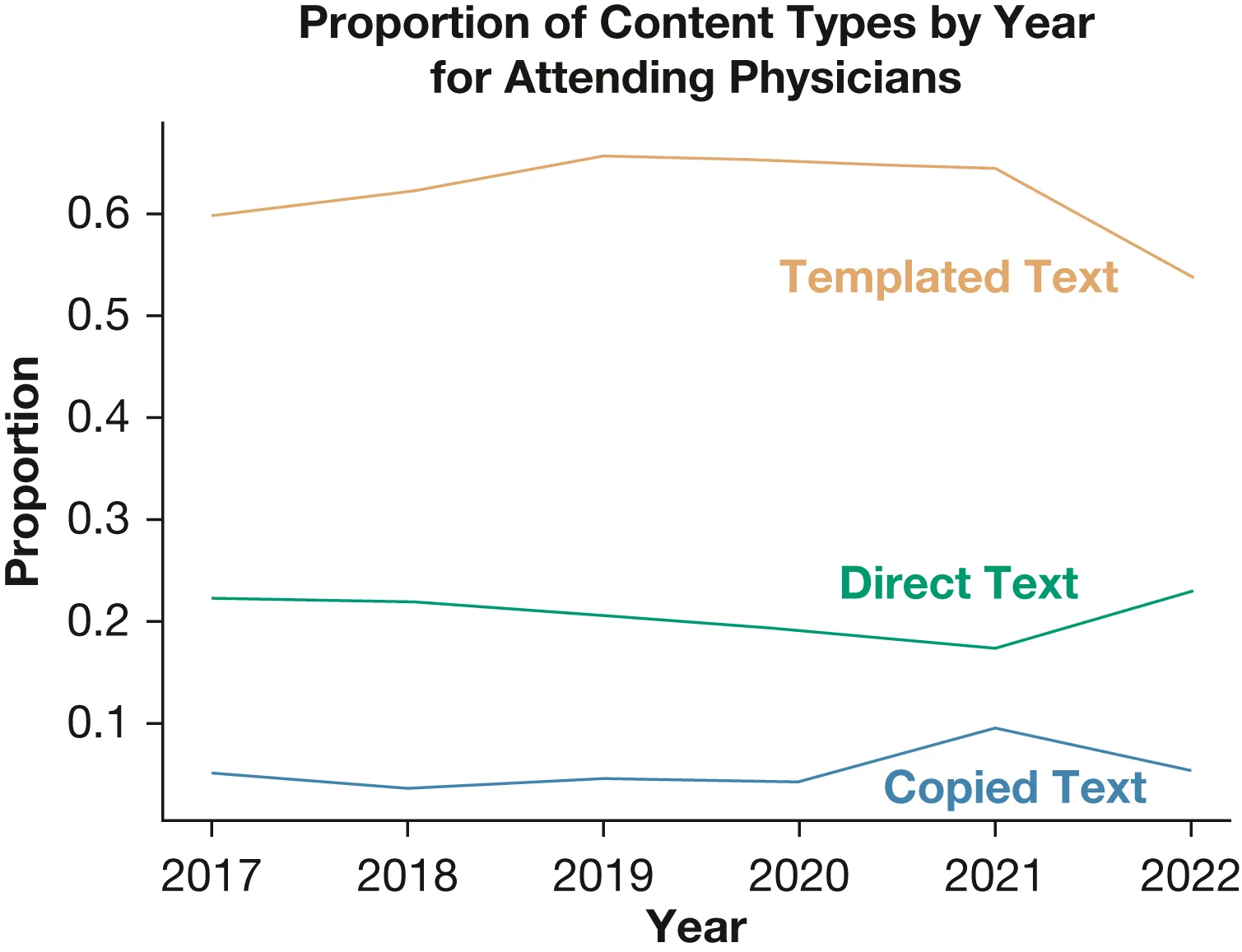
Proportion of copied, direct, or templated text in notes written by attending physicians. There is no statistically significant change in the proportion of copied text, whereas direct text proportion has increased and templated text proportion has decreased.

In attending-written notes, the proportion of copied text remained unchanged (5% vs 5%), whereas direct text increased slightly (22% to 23%; relative increase of 5%; *P* < .01) and templated text declined (60% to 54%; relative decrease of 10%; *P* < .001) (Fig 3). Total note length was positively correlated with both copied text (ρ = 0.22; *P* < .001) and templated text (ρ = 0.42; *P* < .001), and negatively correlated with direct text (ρ = −0.55; *P* < .001).

**Figure 4:**
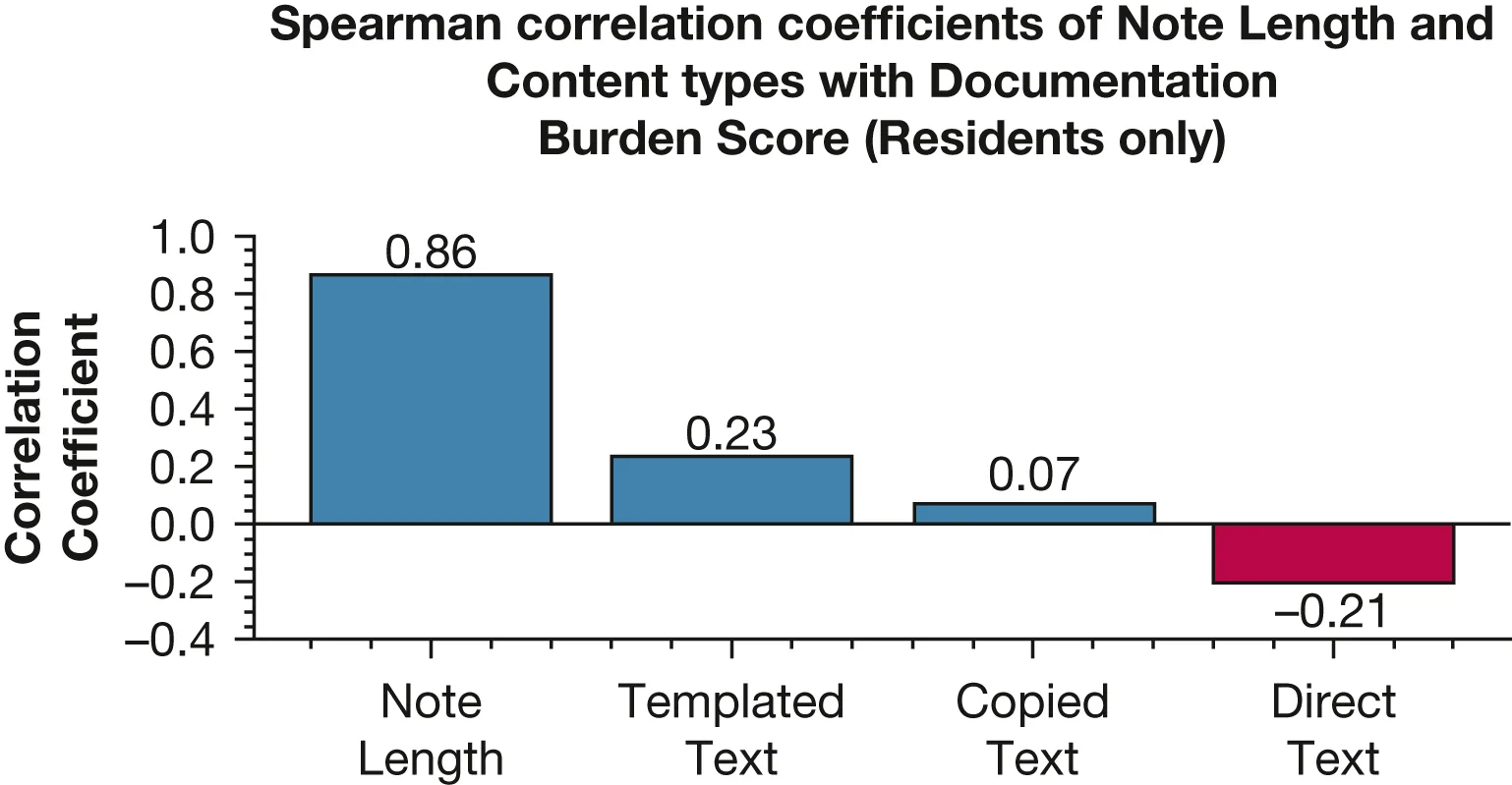
Correlation of documentation burden score with total note length and proportion of copied, templated, or manually entered characters for resident physicians.

The estimator of documentation burden was positively correlated with proportion of templated text (ρ = 0.23; *P* < .001) and copied text (ρ = 0.07; *P* < .001), and negatively correlated with direct text (ρ = −0.21; *P* < .001) (Fig 4). This estimator was positively correlated with total note length (ρ = 0.86; *P* < .001).

We manually reviewed 180 randomly selected resident notes to assess the impact of text duplication on error creation and documentation accuracy. Seven charts (4%) were excluded from the final analysis due to the patient leaving the MICU on the day the progress note was written. We were unable to determine the precise time of care handoff, thus precluding the accurate attribution of errors to the appropriate service. These manually reviewed notes were similar in length and structure to the parent cohort. The median note length was 11,494 characters (IQR, 9,236-14,899). The proportions of copied, templated, and direct text were 55% (IQR, 42%-67%), 35% (IQR, 25%-46%), and 8% (IQR, 5%-14%), respectively. All notes contained copied text, with 79 notes (46%) containing text from another author. The physical examination contained copied text in 131 progress notes (76%), with 44 notes (25%) partly composed and 18 notes (10%) completely composed of content from another author. All assessment and plans contained copied text, again with 79 (46%) containing some portion of text copied from a different author.

**Figure 5:**
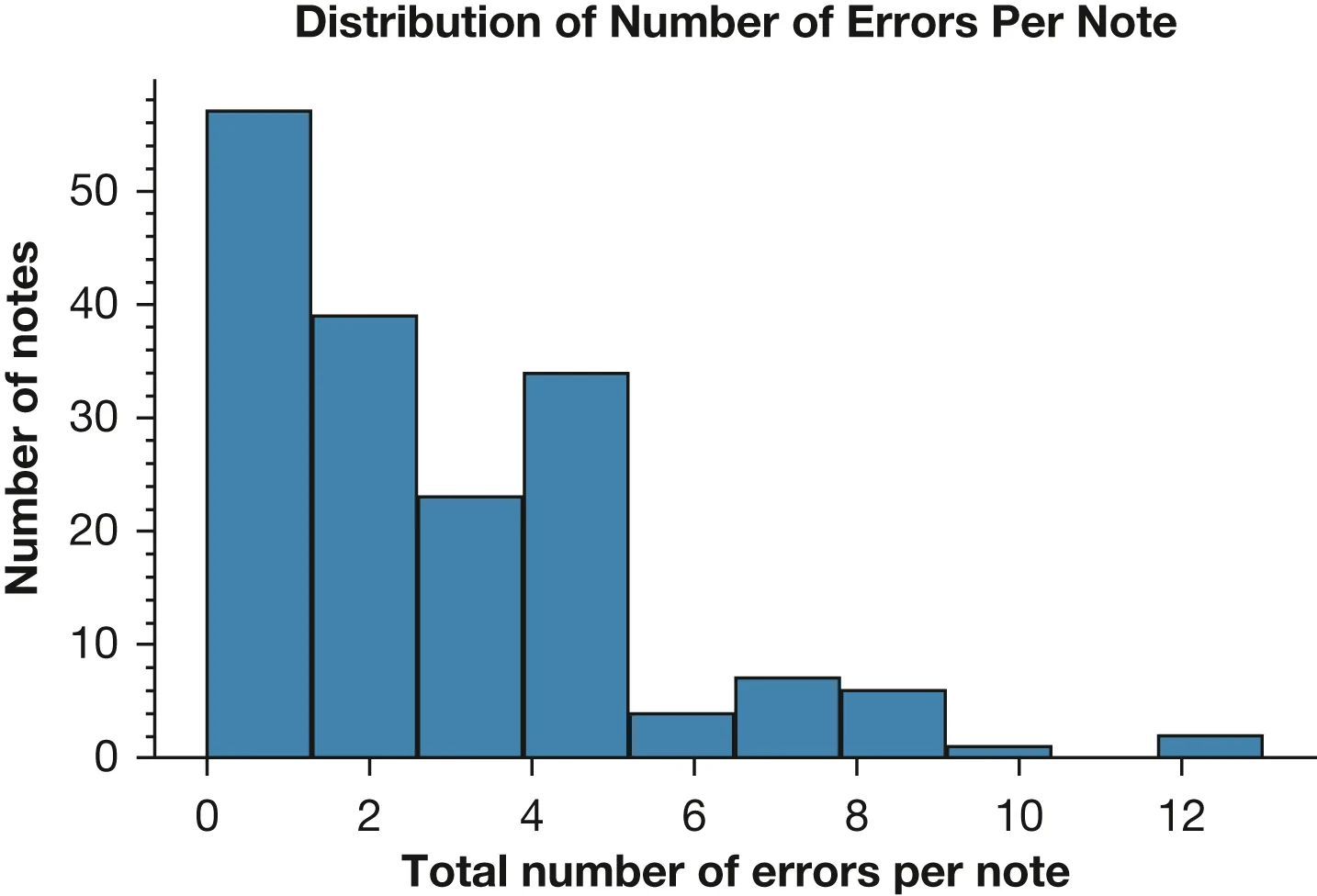
Distribution by number of errors per note. On average, there were about 3 errors per note that were due to copied text (n = 173).

**Figure 6:**
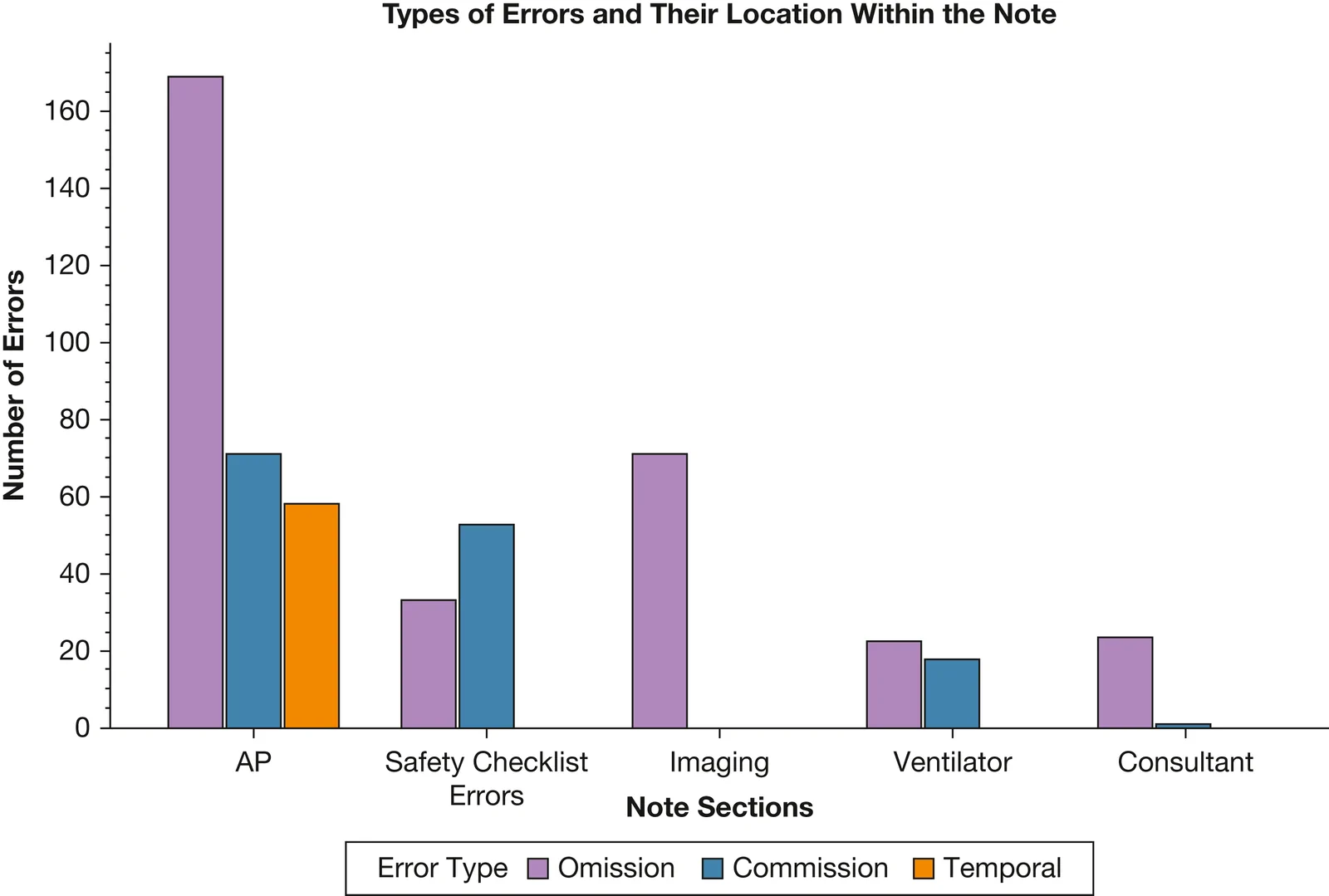
Error types and the section they were found in the note. The AP section of the note contained the largest number of errors (n = 173). AP = assessment and plan.

Review of copied text against gold standards identified 521 errors in 149 notes (86%), averaging roughly 3 copy-paste errors per note (Fig 5). There were 320 errors of omission (61%), 143 errors of commission (28%), and 58 temporal errors (11%). Most errors occurred in the assessment and plan (n = 450, 86%), followed by imaging review (n = 71, 14%). Errors within the assessment and plan were further subdivided and showed 41 errors (8% of total) in ventilator-specific orders or documentation, 25 errors (5% of total) in review of consultant recommendation, and 86 errors (17% of total) in the Safety Checklist (ie, FASTHUG checklist^27^). Recent laboratory results were imported via templated text in all notes and did not contain any copied text; thus, this section was not reviewed for errors. The distributions of errors by type and location within the chart can be seen in Figure 6.

Finally, we assessed associations between note content type, error rate, and clinical encounter measures. Total error count was not associated with total hospital length of stay, elapsed hospital length of stay, nor total note length. Evaluating error subtypes revealed a positive correlation between temporal errors and elapsed length of stay (ρ = 0.15; *P* < .05), and proportion of copied text (ρ = 0.22; *P* < .01), but a negative correlation with templated text proportion (ρ = −0.19; *P* < .01) and direct text proportion (ρ = −0.18; *P* < .05). Neither errors of commission nor errors of omission correlated with elapsed hospital length of stay, total hospital length of stay, total note length, or proportion of content type.

## Discussion

This study provides a detailed analysis of how EHR adoption and its documentation features have reshaped MICU note length and structure. In our closed MICU where residents do most documentation, resident-written notes grew to a median length of 10,690 characters, equivalent to 3.5 single-spaced pages written per patient per day. Conversely, attending notes increased only marginally with a median note length of 1,922 characters. Despite this dichotomy between resident and attending note length, longer notes from either author type were associated with a greater proportion of copied content. Also of interest was that periods of higher documentation burden exacerbated note length; however, this was driven more by higher proportions of templated content and, to a lesser extent, copied content. This finding appears to contradict Kahn et al,^28^ who demonstrated that templates could effectively reduce note bloat. However, their intervention used expressly designed templates and concomitant training to limit autopopulation of large swaths of text. Our findings suggest that in workflows where such targeted design and training are absent, the default use of passive text generation tends to increase note length. This behavior may be explained from the perspective of cognitive load theory, where clinicians work under high levels of intrinsic load and seek to reduce their immediate germane load by using passive tools like copy-paste and templates to circumvent the demanding task of information synthesis. This practice, however, may paradoxically increase downstream extraneous load for subsequent readers as notes lose their cohesiveness or become excessively long.

Perhaps more concerning was that 521 errors resulting within copy-pasted text were found, affecting 86% of notes and translating into roughly 3 copy-paste related errors per note. This error rate is 3 times higher than that found in a previous study.^10^ Prior literature has warned that copied content causes a loss of detail and fidelity within the clinical narrative, exacerbates information chaos, introduces errors, and can be associated with patient harm.^11^, ^12^, ^13^, ^14^, ^15^^,^^29^ Consistent with these concerns, our study confirms a high prevalence of copy-paste errors, perhaps more so than previously measured. When trying to assess how these errors were affected by copy-paste, only the rate of temporal-type errors was positively correlated with the proportion of copied text and negatively correlated with templated text.

To our knowledge, this is the first study to examine ICU documentation behavior changes associated with the adoption of modern EHRs. This study’s findings of increasing note length and growing proportion of duplicated content are consistent with other research conducted in outpatient or other inpatient settings.^5^, ^6^, ^7^, ^8^, ^9^, ^10^ These trends described are concerning for several reasons. First, documentation errors are a major contributor to impaired clinician decision-making and diagnostic error.^16^^,^^17^^,^^29^^,^^30^ Such errors can arise from loss of detail and fidelity in the clinical narrative, the introduction of information chaos and note bloat, and further propagation verbally during multidisciplinary rounds.^11^, ^12^, ^13^^,^^31^^,^^32^ These impediments to accurate clinical decision-making may be exacerbated in the ICU, where higher clinical acuity shortens decision-making time and increases the potential for error and patient harm. Documentation errors have been directly implicated in settled medical malpractice cases.^33^ Moreover, with the advent of artificial intelligence-assisted documentation relying on large language models, there is growing concern that copy-paste practices and their associated errors may poison models and introduce new forms of error creation and propagation.^34^^,^^35^

Interestingly, although copy-paste was initially heralded as a way to accelerate clinical documentation and potentially improve accuracy, studies suggest that these benefits may not be fully realized.^36^ Research in ambulatory care indicates that copy-pasting does not improve time to chart closure and may even be associated with delays in chart completion or increases in after-hours documentation.^37^, ^38^, ^39^, ^40^ Our study further confirms several negative impacts associated with copy-paste use. Providers are aware of the tension between the risks and benefits of copy-paste: although acknowledging that it introduces errors and reduces note quality, many thought the net benefits still outweigh the risks and are reluctant to lose this tool.^36^^,^^41^ Our study provides a descriptive analysis of documentation practices in a high-acuity setting and seemingly confirms this tension because we see continued use of copy-paste despite its association with documentation errors. Although banning copy-paste has been shown to reduce hospital readmissions, it has not proven to be a durable solution to curbing its use.^14^^,^^15^

Instead, sustainable improvements require measurement of these trends and associations to inform solutions or replacements for copied text—or for passively generated content more broadly—that address both provider perceptions and the underlying behavioral drivers. Prior work to combat errors and bloat required both training and institutional templates, emphasizing the need for a multipronged approach.^28^ However, a single didactic education session regarding the hazards of copy-paste may be insufficient to counteract the daily pressure to prioritize speed over precision. Our institution has deployed in-depth simulation-based training at multiple points in a provider’s career, and these could be leveraged to provide the educational component to improve documentation practices. Simulated cases could mirror the types of errors identified in this study and demonstrate to end users the patient safety risks in a controlled environment.

Furthermore, these educational efforts must be reinforced by structural changes in how institutions monitor quality.^42^ As suggested by our study, there is a significant opportunity to integrate documentation integrity into broader quality assurance frameworks. Currently, quality assurance reviews often focus on the existence of documentation for billing compliance rather than its clinical accuracy. Integrating validated tools (eg, modified 9-item Physician Documentation Quality Instrument^43^) could allow institutions to quantify degradation in ICU note quality and better characterize the relationship between copy-paste practices and errors in care delivery. A more robust approach would include these assessments of copy-paste misuse as a standard component of a root cause analysis for adverse events. If an error in care delivery can be traced back to a documentation error in copied text, this link should be explicitly captured to drive systemwide awareness. Additionally, institutions should consider providing caregivers with individual feedback on their documentation habits. Documentation composition metrics (eg, proportion of copied content) could be provided alongside other standard metrics to aid awareness of behaviors that could introduce errors and risk.

Finally, new technology may provide alternative solutions to the manual, keyboard-reliant generation of text. The emergence of ambient scribes driven by artificial intelligence offers a promising method to reduce documentation burden and mitigate the need for copy-paste entirely. However, the ICU presents unique challenges for these tools, characterized by multidisciplinary verbal rounds, increased background noise, and frequent interruptions. Their effectiveness and safety in high-acuity settings remains to be established, and we are currently working to validate these developing tools.^44^ Ultimately, health care systems must move beyond passive acceptance of these tools and actively engineer environments that prioritize documentation accuracy alongside efficiency.

### Study Limitations

Several factors likely contributed to an underestimation of errors. Due to the retrospective nature of this study, it was not possible to verify certain portions of the note (eg, patient’s subjective complaints that day because there is no other documented reference standard). Additionally, there is currently no automated methodology for cross-referencing statements made within a note against primary clinical data, making comprehensive computational evaluation of the entire corpus challenging. As such, our scope was limited to what could be feasibly completed through manual chart review, which likely led to a further underestimation of errors. Conversely, due to reviewing orders in the 24-hour period before note finalization, we may be overcounting errors if an order was placed shortly after the note was signed. Finally, Epic’s methodology for tracking authorship over successive notes may create a risk of misattribution because it may credit an intermediate author rather than tracing it back to an initial author.

An important outstanding question is how patients and other consumers of clinical notes (eg, consultants) are affected by the factors described in this study. Prior studies have found approximately 20% of patients identify errors in their own medical record, and these errors can be associated with harm.^19^^,^^33^ However, more subtle forms of clinical impact—such as how information scatter or obfuscation introduced by copy-paste affects the workflows of consultants or ancillary services—are difficult to assess. This study did not address the clinical significance of copy-paste and related errors, and this represents an important area for future research.

Finally, although we prioritized replicable metrics to facilitate interinstitutional comparison, we concede that there are many drivers of note length beyond our control. We could not fully account for confounders such as patient complexity, severity of illness, or author training level because these data are not structured EHR data. Although attempts were made to control for some differences in documentation behavior at the individual level, the omission of other potential confounders limits our ability to attribute increasing note length to a specific driver.

## Interpretation

In this study, we provide a detailed characterization of content sources and their impact on note length and documentation accuracy. We found that notes are increasing in length, driven largely by passively generated content sources such as copied or templated text. In resident-written notes, the largest source of content was copied text (50% of all characters), whereas attendings relied more heavily on templated text (57% of characters). Among residents, usage patterns shifted with changes in their daily documentation burden; higher burden led to increased reliance on copied and templated text and decreased use of direct text. The most common error type within copied text was omission (61%). Only temporal errors were significantly associated with the proportion of copied text. These findings suggest that the interplay between content sources and documentation accuracy is complex and may help explain some sources of documentation error in the current era of provider-entered clinical records.

## Funding/Support

R. G. is a recipient of the National Library of Medicine’s T15 Biomedical Informatics and Data Science Research Training grant [Grant T15 LM 7088-33]. J. A. G. is the primary investigator for Agency for Healthcare Research and Quality (AHRQ) Grant R18 HS029345-01.

## Financial/Nonfinancial Disclosures

None declared.
